# A Single Amino Acid Change Converts the Sugar Sensor SGLT3 into a Sugar Transporter

**DOI:** 10.1371/journal.pone.0010241

**Published:** 2010-04-20

**Authors:** Laura Bianchi, Ana Díez-Sampedro

**Affiliations:** Department of Physiology and Biophysics, Miller School of Medicine, University of Miami, Miami, Florida, United States of America; Brigham and Women's Hospital, Harvard Medical School, United States of America

## Abstract

**Background:**

Sodium-glucose cotransporter proteins (SGLT) belong to the *SLC5A* family, characterized by the cotransport of Na^+^ with solute. SGLT1 is responsible for intestinal glucose absorption. Until recently the only role described for SGLT proteins was to transport sugar with Na^+^. However, human SGLT3 (hSGLT3) does not transport sugar but causes depolarization of the plasma membrane when expressed in *Xenopus* oocytes. For this reason SGLT3 was suggested to be a sugar sensor rather than a transporter. Despite 70% amino acid identity between hSGLT3 and hSGLT1, their sugar transport, apparent sugar affinities, and sugar specificity differ greatly. Residue 457 is important for the function of SGLT1 and mutation at this position in hSGLT1 causes glucose-galactose malabsorption. Moreover, the crystal structure of *vibrio* SGLT reveals that the residue corresponding to 457 interacts directly with the sugar molecule. We thus wondered if this residue could account for some of the functional differences between SGLT1 and SGLT3.

**Methodology/Principal Findings:**

We mutated the glutamate at position 457 in hSGLT3 to glutamine, the amino acid present in all SGLT1 proteins, and characterized the mutant. Surprisingly, we found that E457Q-hSGLT3 transported sugar, had the same stoichiometry as SGLT1, and that the sugar specificity and apparent affinities for most sugars were similar to hSGLT1. We also show that SGLT3 functions as a sugar sensor in a living organism. We expressed hSGLT3 and E457Q-hSGLT3 in *C. elegans* sensory neurons and found that animals sensed glucose in an hSGLT3-dependent manner.

**Conclusions/Significance:**

In summary, we demonstrate that hSGLT3 functions as a sugar sensor *in vivo* and that mutating a single amino acid converts this sugar sensor into a sugar transporter similar to SGLT1.

## Introduction

SGLT1 is an electrogenic transporter that couples the movement of two Na^+^ ions to the transport of a single sugar molecule into cells [1]. Its biophysical and physiological properties are well characterized, leading to detailed structural and mechanistic models of its function [2]. Human SGLT3 (hSGLT3) is a protein from the same family as SGLT1 that despite a high degree of amino acid identity appears to have different characteristics. First, hSGLT3 does not transport sugars, although sugars bind to the protein. The consequence of sugar binding to hSGLT3 is membrane depolarization resulting from cation permeation [3]. In addition, sugars (except imino sugars) bind hSGLT3 with much weaker apparent affinity than hSGLT1 [4]. Finally, hSGLT3 has different sugar selectivity than hSGLT1 [4]. For example, hSGLT3 binds imino sugars while hSGLT1 does not. Because the binding of sugars to hSGLT3 causes membrane depolarization and not sugar transport, it has been suggested that hSGLT3 functions as a sugar sensor instead of a sugar transporter [3].

hSGLT3 is expressed at the neuromuscular junction and in the enteric nervous system. In both cases hSGLT3 colocalizes with the acetylcholine receptor [3]. These data suggest that sugar sensing by hSGLT3 may occur in multiple tissues. Other studies have suggested that sugar sensing may be a common function of SGLT proteins [5], [6].

**Figure 1:**
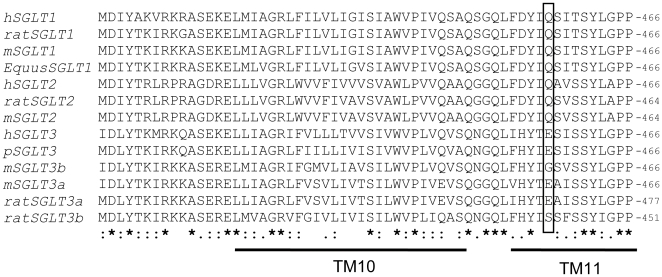
Amino acid alignment of SGLTs. Four SGLT1s and 3 SGLT2s from different species (h: human, m: mouse, p: pig) have a glutamine at position 457. SGLT1s and SGLT2s proteins transport sugars. The alignment also includes 6 SGLT3s. None of the SGLT3s encodes a glutamine at position 457 (boxed). Rather a glutamate, glycine or serine is present at that position. In SGLT1, Q457 is involved in sugar recognition, binding and translocation [8], [9] and mutation at this site can cause glucose-galactose malabsorption [2], [7]. Stars, semicolons and periods denote identity and conservation respectively. Transmembrane segments (TM) 10 and part of 11 are also shown (based on alignment with vSGLT, whose crystal structure has been solved [9]).

Residue Q457 in transmembrane segment 11 of hSGLT1 plays a key role in its function. The importance of this residue was initially revealed by the finding that a patient with glucose-galactose malabsorption, a disease characterized by inability to absorb intestinal glucose, had a mutation at this site (Q457R) [2], [7]. Subsequent structure-function studies revealed that residue Q457 in hSGLT1 is likely involved in sugar binding and translocation through hydrogen bond interactions with the pyranose ring of the sugar [8]. More recently, the crystal structure of the *vibrio* SGLT (vSGLT) shows that the residue corresponding to amino acid 457 in mammalian proteins directly interacts with the sugar [9]. Interestingly, while all cloned SGLT1 and SGLT2 cotransporters have a conserved glutamine at amino acid 457, SGLT3s have either glutamate (human SGLT3, pig SGLT3, rat SGLT3a and mouse SGLT3a), glycine (mouse SGLT3b) or serine (rat SGLT3b) (Fig. 1).

It is not known if amino acid 457 underlies the striking SGLT1/SGLT3 functional differences. Specifically, we wondered if introducing a glutamine instead of a glutamate at position 457 in hSGLT3 (E457Q-hSGLT3) would transform hSGLT3 into a sugar transporter. Moreover, despite finding that hSGLT3 acts like a sugar sensor in *Xenopus* oocytes, there is no direct evidence that it acts as a sugar sensor *in vivo*. In this paper we report that substitution of E457 with a glutamine converts hSGLT3 into a sugar transporter with functional features resembling SGLT1, including lower K_0.5_s for sugars and modified sugar specificity. We also show that hSGLT3 functions as a sugar sensor *in vivo* when expressed in *C. elegans* ASK chemosensory neurons.

## Results

### A point mutation converts hSGLT3 from a sugar sensor to a sugar cotransporter

**Figure 2:**
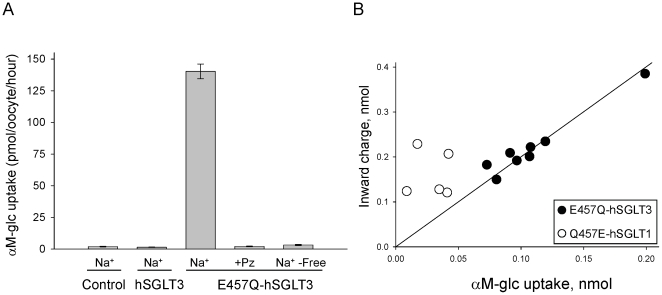
Substitution of glutamate by glutamine at position 457 in hSGLT3 changes the protein from a glucose sensor to a glucose transporter. **A.** We measured 50 µM αM-glc transport in control oocytes (non-injected) and WT-hSGLT3 and E457Q-hSGLT3 expressing oocytes. Data show the mean ± SE of the sugar uptake in individual oocytes. The number of oocytes per condition varied from 4 to 8. In oocytes expressing E457Q-hSGLT3, experiments were also carried out in the presence of 0.1 mM phlorizin (Pz), or in absence of Na^+^. **B.** Charge uptake and sugar uptake were simultaneously measured in oocytes expressing E457Q-hSGLT3 (filled circles) and Q457E-hSGLT1 (open circles). Each point corresponds to datum obtained from one oocyte clamped in the presence of αM-glc. The background uptake of αM-glc in non-injected oocytes has been subtracted. The solid line shows a fit of E457Q-hSGLT3 data with a linear regression giving a slope of 2.0 indicating that 2 net positive charges are transported per αM-glc molecule.

In spite of the high amino acid identity between hSGLT3 and hSGLT1 (70%), the biophysical characteristics of the proteins differ greatly. hSGLT3 has a glutamate at position 457 while all SGLT1 sugar transporters have a glutamine (Fig. 1). Previous studies on SGLT1 [8] and the recently resolved structure of vSGLT [9] suggest that the amino acid at positions corresponding to 457 in SGLT1 plays a direct role in sugar binding and transport. We investigated the functional consequences of substituting E457 to glutamine in hSGLT3. To study the function of E457Q-hSGLT3, ^14^C-αM-glc (alpha-methyl-D-glucose, a non-metabolizable sugar that is a substrate for SGLT proteins) transport experiments were carried out in *Xenopus* oocytes expressing hSGLT3 or E457Q-hSGLT3 (Fig. 2A). As expected from previous studies, hSGLT3 expressing oocytes did not transport sugar [3]. However, E457Q-hSGLT3 expressing oocytes showed a dramatic increase in sugar uptake, transporting approximately 100 times more sugar than non-injected (control) or hSGLT3 expressing oocytes. To confirm that sugar uptake was through E457Q-hSGLT3, we repeated the experiments in the presence of 0.1 mM phlorizin (Pz), a competitive inhibitor of SGLT proteins, or in the absence of Na^+^, since SGLT proteins use the Na^+^ electrochemical gradient to drive sugar transport. In these two conditions, the sugar uptake was as low as in non-injected oocytes. These results show that sugar transport in E457Q-hSGLT3 expressing oocytes is Na^+^ dependent and is inhibited by Pz, indicating that it is mediated by the E457Q-hSGLT3. These data demonstrate that mutating E457 to Q transforms the sugar sensor hSGLT3 into a sugar transporter.

SGLT1 uses the electrochemical gradient of Na^+^ to transport sugar. In each transport cycle the stoichiometry is 2 Na^+^/1 glucose [1]. We thus explored whether a similar stoichiometry existed in E457Q-hSGLT3. We recorded current induced by 1 mM αM-glc in individual oocytes expressing E457Q-hSGLT3, clamped at −80 mV, and simultaneously measured ^14^C-αM-glc transported into cells (filled circles in Fig. 2B). We fitted a linear regression to the data points and found that the stoichiometry was 2.0 positive charges transported per αM-glc molecule, the same as in SGLT1, indicating that ion and sugar transport in E457Q-hSGLT3 is tightly coupled and neither ions nor sugar leak through the protein. This suggests that the side chain at 457 in SGLT proteins is responsible for coupling ion flux to sugar transport. To test this, we performed the same experiment in the hSGLT1 mutant Q457E, which replaces the amino acid in hSGLT1 for that in hSGLT3. We previously reported that the K_0.5_ for sugars in Q457E-hSGLT1 is weaker than in WT-hSGLT1 [8]. Here we show that Q457E-hSGLT1 behaved as an uncoupled transporter (empty circles, Fig. 2B). These data clearly indicate the importance of residue 457 on the coupling between ions and sugar, and the resulting stoichiometry.

### E457Q mutation increases the sugar-induced currents

**Figure 3:**
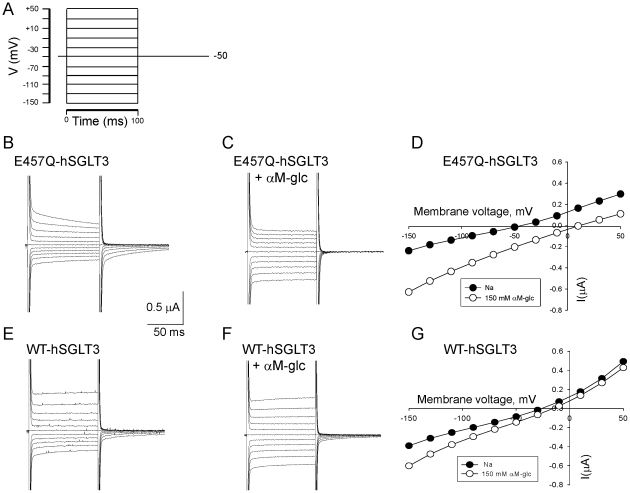
Current recordings in WT and in E457Q-hSGLT3. **A.** Voltage/pulse protocol used in experiments shown in panels B–G. The voltage is initially clamped at −50 mV, then the voltage jumped from −150 to +50 mV with 20 mV increments for 100 ms and finally the voltage is returned to -50 mV. **B.** Current recordings in a E457Q-hSGLT3 expressing oocyte in presence of Na^+^. **C.** Analogous currents are shown from the same oocyte in the presence of 150 mM αM-glc. Note that the presteady-state currents disappear after adding sugar. **D.** Steady-state currents in a E457Q-hSGLT3 expressing oocyte at different voltages (from −150 to +50 mV) in Na^+^ alone and after adding 150 mM αM-glc. **E, F, and G.** Currents recorded with the same conditions as in A, B, and C respectively in a WT-hSGLT3 expressing oocyte. Note that the presteady-state currents in E457Q-hSGLT3 (B) are larger than in WT-hSGLT3 (E).

In order to further study the characteristics of E457Q-hSGLT3, we recorded sugar-induced currents. We clamped the oocyte at −50 mV and measured currents at voltages ranging from −150 to +50 mV in 20 mV decrements (Fig. 3A) in the absence (Fig. 3B) and presence (Fig. 3C) of 150 mM αM-glc. The current/voltage relationships obtained from these data are shown in Figure 3D. In Figure 3E, F and G data obtained from an oocyte expressing WT-hSGLT3 are shown for comparison. Our data show that the sugar-induced current (difference between black and open circles in 3D and 3G) is larger in E457Q-hSGLT3 than in WT-hSGLT3 expressing oocytes.

In the absence of sugar, SGLT1 proteins exhibit pre-steady-state currents after a step change in membrane voltage. These currents are generally attributed to sodium binding and voltage-dependent changes in the conformation of the transporter [10], [11]. The pre-steady-state currents in WT-hSGLT3 were very small compared to those reported for SGLT1. Figure 3B shows that the pre-steady-state currents were larger in E457Q-hSGLT3 (Fig. 3B) than in WT-hSGLT3 (Fig. 3F). The pre-steady-state currents disappeared completely when sugar was added to the mutant (Fig. 3C), but only decreased slightly when sugar was added to WT-hSGLT3 (Fig. 3F). It is possible that the lower apparent affinity of the wild-type protein for this sugar leaves a greater fraction of SGLT3 proteins unbound to sugar.

To confirm that these results were due to the activity of WT-hSGLT3 and E457Q-SGLT3 we performed the same experiments in non-injected oocytes (Figure S1). Pre-steady-state currents were not present in non-injected oocytes (Fig. S1A) and 150 mM αM-glc did not induce current (Fig. S1C).

### E457Q mutation changes hSGLT3 ligand specificity and apparent affinity of sugars

**Figure 4:**
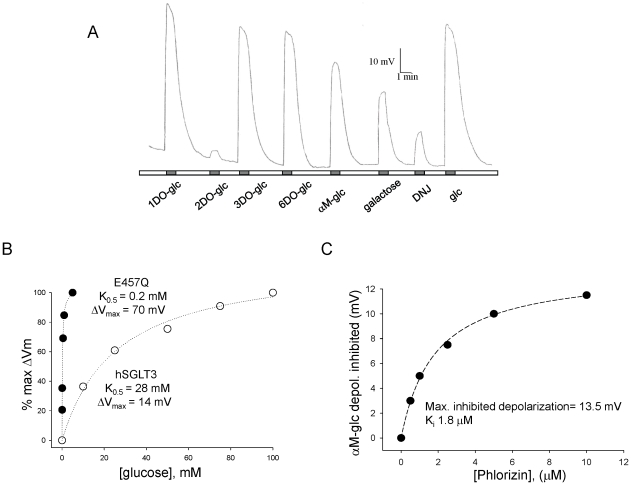
Apparent affinities in E457Q-hSGLT3 are similar to hSGLT1 affinities. **A.** Depolarizations induced by different sugars in an E457Q-hSGLT3 expressing oocyte. When used at saturating concentrations, different sugars induced similar maximal depolarizations. The concentrations were 500 µM for DNJ; 10 mM for 1DO-glc, glc and αM-glc; 50 mM for 2DO-glc, 3DO-glc, 6DO-glc; and 100 mM for galactose. Galactose showed a smaller depolarization because the concentration tested was not saturating. 2DO-glc and DNJ showed small depolarizations and are known to be poor hSGLT1 agonists. **B.** Depolarizations induced by glucose in one representative oocyte expressing E457Q-hSGLT3 and one hSGLT3 are shown as a function of sugar concentration. Dotted lines represent the fit of the data with equation (1) to estimate the K_0.5_ and maximal depolarization (ΔV_max_) values. K_0.5_ in this example was 0.4±0.1 mM for E457Q-hSGLT3 and 28±3 mM for hSGLT3. **C.** K_i_ of phlorizin for E457Q-hSGLT3. Graphic illustrates the inhibition of 0.5 mM αM-glc-induced depolarization by phlorizin. The amount of αM-glc-induced depolarization inhibited by 0–10 µM Pz is plotted against Pz concentration. The curve is a fit to equation (1) (Methods).

We next tested whether E457Q imparted other SGLT1-like properties to SGLT3. We first recorded sugar-induced depolarizations in E457Q-hSGLT3 expressing oocytes and compared our results with published data for WT-hSGLT3 and hSGLT1 [4]. Figure 4A shows that five of the eight sugars we tested [1-deoxy-glucose (-DO-glc), 3DO-glc, 6DO-glc, glc, αM-glc] induced similar maximal depolarizations at saturating concentrations. These data indicate that these sugars interact with E457Q-hSGLT3 with comparable efficiency, and that they are similarly effective as agonists. However, two of the sugars, 2DO-glc and 1-deoxynojirimycin (DNJ), induced much smaller depolarizations indicating poor or lack of interaction with E457Q-hSGLT3. 2DO-glc is neither a substrate for hSGLT1 nor hSGLT3, and DNJ was previously shown to be good ligand for hSGLT3 but a poor one for hSGLT1 [4]. Our data suggest that the E457Q substitution changes the transporter interaction with DNJ and renders it more similar to hSGLT1.

It was previously shown that all sugars and inhibitors tested (except imino sugars) have weaker apparent affinity (K_0.5_) for hSGLT3 than for hSGLT1 [4]. We tested whether the mutation we introduced (E457Q) would change hSGLT3 sugar K_0.5_s to render them similar to hSGLT1. For all sugars in Fig. 4A except 2DO-glc and DNJ the K_0.5_s were then calculated (Table 1). Figure 4B shows one example of depolarization caused by glucose (black circles) at substrate concentrations that range from 0.1 to 5 mM in a representative oocyte. The fit that provides the K_0.5_ value is shown as a dotted line. For comparison we also show the fit obtained from glucose-induced depolarizations in a WT-hSGLT3 expressing oocyte and the fit gives a K_0.5_ similar to that previously published [3], [4]. The results (Table 1) show that substituting glutamate for a glutamine at position 457 not only converted hSGLT3 from a sugar sensor to a sugar transporter, but also dramatically increased the apparent affinities for glucose, αM-glc, 1DO-glc, 3DO-glc and 6DO-glc, so that they are similar or even stronger than those of SGLT1. The only apparent affinity that was not affected by the E457Q mutation was that for galactose (134±22 mM, n = 2), suggesting that E457Q-hSGLT3 retains selectivity for glucose derivatives over galactose derivatives, just like WT-hSGLT3 and in contrast to hSGLT1. Apparent affinities of 2DO-glc and the imino sugar DNJ for E457Q-hSGLT3 were not determined because their induced depolarizations were too small.

**Table 1:** Apparent affinities are similar in E457Q-hSGLT3 and hSGLT1.

	hSGLT1		WT-hSGLT3		E457Q-hSGLT3	
	K_0.5_ (mM)	Ratio^glc^	K_0.5_ (mM)	Ratio^glc^	K_0.5_ (mM)	Ratio^glc^
αM-glc	0.7±0.04^a^	1.4	21±6^c^	1.1	0.5±0.03 (3)	1.7
Glucose	0.5±0.02^a^	1	19±6^c^	1	0.3±0.07 (3)	1
Galactose	0.6±0.02^a^	1.2	ND^c^		134±22 (2)	447
1DO-glc	10±1^a^	20	43±10^c^	2.2	0.3±0.04 (3)	1
2DO-glc	ND^a^		ND^c^		ND	
3DO-glc	ND^a^		ND^c^		2.2±0.2 (3)	7.3
6DO-glc	3±0.5^a^	6	>50^c^		2.9±0.4 (3)	10
DNJ	ND^a^		0.004±0.001^c^	2×10^−4^	ND	
Phlorizin (Ki)	0.0002±0.00001^a^	4×10^−4^	0.12^c^	6×10^−3^	0.0016±0.00009 (3)	5×10^−3^

K_0.5_s were obtained for different sugars in E457Q-hSGLT3 expressing oocytes. Data from WT-hSGLT3 and hSGLT1 are from other studies and are shown for comparison. Next to each K_0.5_ we show the ratio of the K_0.5_ of each sugar compared to glucose. E457Q-hSGLT3 data are mean ± SE in mM obtained from 2–3 oocytes; a =  reference [8]; b =  reference [12]; c =  reference [4]. ND =  not determined (due to low currents).

Phlorizin, in addition to inhibiting SGLT1 with an inhibition constant, K_i_, of 0.2 µM [12] also inhibits hSGLT3 with a K_i_ of 120 µM [4]. We asked if the E457Q mutation also affected phlorizin inhibition of hSGLT3. To calculate the K_i_ of phlorizin for E457Q-hSGLT3, we measured the inhibitory effect of phlorizin at concentrations ranging from 0.1 to 10 µM on depolarizations induced by the K_0.5_ of αM-glc, 0.5 mM, and plotted the inhibition as a function of phlorizin concentration (Fig. 4C). By fitting these data with equation (1) (Methods), we obtained the K_i_ of phlorizin for E457Q-hSGLT3. We found that phlorizin inhibits E457Q-hSGLT3 with an inhibition constant (K_i_  = 1.6 µM) that is stronger than WT-hSGLT3 (Table 1). This observation suggests that E457Q alters the phlorizin binding site or the allosteric coupling of phlorizin binding with the inhibited state. We conclude that E457Q mutation renders hSGLT3 sensitivity to phlorizin similar to hSGLT1.

### hSGLT3 mediates glucose-sensing when expressed in *C. elegans* ASK neurons

**Figure 5:**
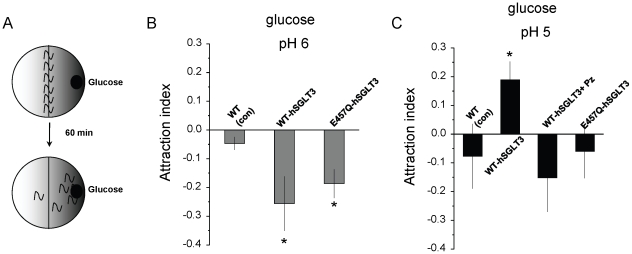
hSGLT3 mediates glucose chemotaxis when expressed in *C. elegans* sensory neurons. **A**. Scheme of the behavioral assay we performed in *C. elegans* (see Methods for details). **B.** Transgenic animals expressing WT-hSGLT3 or E457Q-hSGLT3 in ASK chemosensory neurons were tested for chemotaxis to 10 mM glucose on pH 6 plates in which a gradient of glucose was established. Attraction index (AI) was (number of animals at glucose spot - number of animals at control spot)/(total number of animals). Thirty to forty animals were assayed in each trial. Number of trials was 6, 5, and 8 respectively. *C. elegans* expressing WT-hSGLT3 or E457Q-hSGLT3 in ASK sensory neurons were repulsed by glucose. **C.** The same strains were assayed on pH 5 agar plates. For experiments in which we used phlorizin we incubated the chunk of agar in 10 mM glucose plus 0.1 mM phlorizin, prior to establishing the gradient on the plate. *C. elegans* expressing WT-hSGLT3 were attracted to glucose whereas animals expressing E457Q-hSGLT3 showed no preference for glucose over the control spot. Importantly, attraction of WT-hSGLT3 expressing *C. elegans* to glucose was inhibited when Pz was present, confirming that it was mediated by hSGLT3. Number of trials was 10, 14, 5 and 8 for wild type *C. elegans*, WT-hSGLT3, WT-hSGLT3+Pz and E457Q-hSGLT3 respectively. Data are expressed as mean ± SE. * indicates p<0.05 by comparison with wild type non-transgenic control *C. elegans*, by t-test.

hSGLT3 can function as a sugar sensor when expressed in *Xenopus* oocytes [3]. Does hSGLT3 function as a sugar sensor in a living organism? To test this, we expressed WT-hSGLT3 in *C. elegans* ASK chemosensory neurons. We reasoned that if hSGLT3 alters the membrane potential in these sensory neurons when animals are exposed to glucose, hSGLT3 may be able to drive sugar sensing (Fig. 5A). We also expressed E457Q-hSGLT3 to determine if the functional differences between WT and mutant hSGLT3 observed in the oocytes are reflected *in vivo*.

ASK neurons are located in the head of *C. elegans* and participate with other sensory neurons in mediating attraction to lysine as well as avoidance of acidic solutions [13], [14]. The molecular mechanisms underlying this chemosensitivity are not fully understood but involve the DEG/ENaC channel DEG-1 and TRP channel OSM-9 [14], [15]. We generated transgenic animals expressing WT-hSGLT3 or E457Q-hSGLT3 under the control of the ASK specific promoter *srg-8* [16] and assayed attraction to 10 mM glucose on standard chemotaxis plates (Fig. 5A) [13].

We found that while non-transgenic control *C. elegans* were neither repulsed by nor attracted to 10 mM glucose, *C. elegans* expressing WT-hSGLT3 and E457Q-hSGLT3 were repulsed by glucose (p = 0.043 and p = 0.041 respectively, Fig. 5B). In a previous study, it was shown that in hSGLT3 expressing oocytes glucose induces higher currents with increasing concentration of protons [3]. We thus repeated the experiments on chemotaxis plates at pH 5. We found that while transgenic animals that expressed E457Q-hSGLT3 were no longer repulsed by glucose, WT-hSGLT3 expressing *C. elegans* were now attracted to glucose (Fig. 5C). Attraction was clearly mediated by hSGLT3 because it was inhibited by the SGLT inhibitor phlorizin, and it was not observed in wild type control *C. elegans*. To confirm that ASK neurons remained functional in WT-hSGLT3 and E457Q-hSGLT3 animals, we measured attraction to lysine-acetate which is mediated by ASK neurons. *C. elegans* were attracted to lysine in all genetic backgrounds (attraction index  = 0.37±0.06, 0.29±0.05 and 0.31±0.06 for wild type *C. elegans*, WT-hSGLT3 transgenic *C. elegans*, and E457Q-hSGLT3 transgenic *C. elegans,* respectively) indicating that ASK neurons functioned normally.

**Figure 6:**
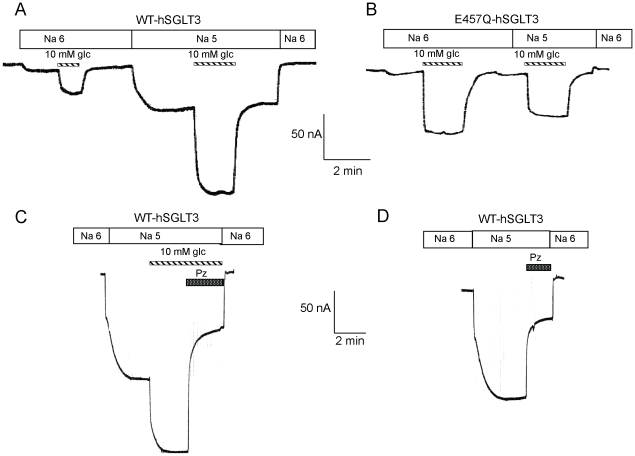
Glucose-induced currents are larger at pH 5 than pH 6 in hSGLT3 expressing oocytes. **A.** At pH 6, 10 mM glucose induced small currents in WT-hSGLT3 oocytes. However, at pH 5 the glucose-induced current increased approximately 4 fold. In addition, there was an inward current when the extracellular solution pH was reduced from 6 to 5. These glucose- or H^+^- induced currents returned to the baseline when the glucose was removed from the bath and when the pH was returned to 6. Striped bars indicate the presence of glucose in the bath. **B.** The same experiment was performed in oocytes expressing E457Q-hSGLT3. Sugar-induced currents were similar at both pHs and H^+^-induced current was absent. **C.** The effect of phlorizin was measured on glucose-induced currents at pH 5 in a WT-hSGLT3 expressing oocyte. Phlorizin blocked approximately two-thirds of the total induced current. **D.** Phlorizin also blocked approximately two-thirds of the current induced by pH 5 in the absence of sugar. These data suggest that phlorizin blocks both the H^+^-induced and sugar-induced components of current.

*C. elegans* expressing WT-hSGLT3 or E457Q-hSGLT3 displayed different behaviors *in vivo* towards glucose depending on the pH. To study how pH affects the function of hSGLT3 and E457Q-hSGLT3, we carried out experiments in oocytes expressing each protein. We clamped the oocytes at −50 mV and measured the sugar-induced current at pH 6 and pH 5 mimicking the conditions of our *C. elegans* experiments. For WT-hSGLT3 (Fig. 6A), we found that 10 mM glucose induced a small current at pH 6. However, when the same oocyte was perfused with pH 5 solution the same concentration of sugar induced a much larger current, approximately 4 times greater. We also observed a H^+^-induced current when the pH was lowered from 6 to 5 even in the absence of sugar. Thus, we show that in oocytes that express WT-hSGLT3 the combination of pH 5 and 10 mM glucose produced much larger currents than glucose at pH 6. In contrast, in E457Q-hSGLT3 expressing oocytes (Fig. 6B), the 10 mM glucose-induced current was almost the same at both pH 6 and pH 5. And the H^+^-induced current observed in WT-hSGLT3 when dropping the pH was almost non-existent in E457Q-hSGLT3. Thus, increasing [H^+^] had a much smaller effect on E457Q-hSGLT3 induced currents than on WT-hSGLT3. Even though low pH increases glucose-induced currents, WT-hSGLT3 still does not transport glucose in these conditions (Fig. S2).

*C. elegans* expressing WT-hSGLT3 were neither repulsed by nor attracted to glucose when 100 µM Pz was present (Fig. 5C). Thus, we also tested how the glucose-induced current was affected by Pz. Figure 5C shows that Pz significantly inhibited the current induced by glucose and H^+^ at pH 5. Indeed Pz inhibits ∼2/3 of the current. To confirm the effect of Pz on the isolated H^+^-induced currents we repeated the experiment in the absence of sugar (Fig. 6D). To conclude, these results show that at the same concentration used in our *C. elegans* experiments, 100 µM, Pz significantly inhibits sugar induced currents at pH 5, thus explaining why there is no attractive effect of glucose on *C. elegans* with the inhibitor. Importantly, our data also indicate that the H^+^-induced current in the absence of sugar is mediated by hSGLT3, as it is inhibited by Pz.

### E457Q mutation decreases the H^+^ permeability in hSGLT3

The proton-induced current observed in WT-hSGLT3 may be carried by either Na^+^ or H^+^ ions. To establish which ion permeates hSGLT3 at pH 5, we calculated the reversal potentials (E_rev_) of WT-hSGLT3 and E457Q-hSGLT3 in oocytes at physiological pH (7.4) and at pH 5 in the presence or absence of Na^+^ (Table 2). In WT-hSGLT3, E_rev_ shifted ∼ +70 mV when extracellular pH was changed from 7.4 to 5 in Na^+^ and without Na^+^. However, in E457Q-hSGLT3, the shift was smaller (+30−40 mV) than in the WT-hSGLT3. For a perfectly selective proton channel a shift of +138 mV is expected for this pH change. These results suggest that both H^+^ and Na^+^ ions permeate through WT-hSGLT3 and E457Q-hSGLT3, but that H^+^ ions permeate more readily through WT-hSGLT3 than through E457Q-hSGLT3. Note that in non-injected oocytes the shift was only ∼ +2 mV indicating that H^+^ ions do not leak across the oocytes' plasma membrane.

**Table 2:** H^+^ permeation is higher in E457Q-hSGLT3 than in WT-hSGLT3.

E_rev_ (mV)	N7	N5	ΔN7-N5	C7	C5	ΔC7-C5
hSGLT3	−39±4	+29±2	+68±5	−29±1	+39±6	+68±7
E457Q-hSGLT3	−46±4	−16±5	+30±6	−52±2	−6±6	+43±3

Reversal potentials (E_rev_) obtained from oocytes expressing WT-hSGLT3 or E457Q-hSGLT3 in the presence of Na^+^ at pH 7.4 (N7) and pH 5 (N5) and in the absence of Na^+^ at the same pHs (C7 and C5). Δ values in both conditions are also shown. In WT-hSGLT3 expressing oocytes, there was a +68 mV shift in the reversal potential when pH was lowered to 5 both in the absence and presence of Na^+^. In contrast, in E457Q-hSGLT3 the shift was smaller (∼30–40 mV). Data are shown as mean ± SEM, n = 4.

These data suggest that in our animal model, more H^+^ ions are likely to enter into ASK neurons in WT-hSGLT3 than E457Q-hSGLT3 transgenic *C. elegans*, leading to greater depolarization. This may explain why *C. elegans* expressing WT-hSGLT3 or E457Q-SGLT3 behave so differently on pH 5 plates containing glucose. At this point it is unclear whether larger depolarizations that result from larger currents at pH 5 in WT-hSGLT3 expressing ASK neurons, or whether the protons themselves, drive attraction to glucose. Regardless of the precise mechanism our data argue that when WT-hSGLT3 or E457Q-hSGLT3 are expressed in *C. elegans* ASK neurons, they are capable of inducing a behavioral response to glucose. This response turns from repulsion to attraction when WT-hSGLT3 currents are enhanced by low pH and currents are carried by both Na^+^ and H^+^.

## Discussion

### Channel or transporter

Because hSGLT3 belongs to a family of transporters and has high amino acid identity with human SGLT1, the Na^+^/glucose cotransporter, the finding that it was a sugar sensor and not a sugar transporter was unexpected [3]. However, the distinction between channels and transporters, once thought to be very clear, has become increasingly blurred. The CLC family is a prime example of this, as CLC-ec was found to be a H^+^-Cl^−^ antiporter [17], and not a channel as predicted. In fact, five of nine members of the family of human ClC proteins are antiporters and not channels [18]–[20]. Another example of this blurred boundary is the Excitatory Amino Acid Transporter 2 (EAAT2), a glutamate transporter which also has a ligand-dependent Cl^−^ channel current [21]. In fact, a recent meeting and review pointed to numerous examples of transporters that show channel-like behavior, either in their wild-type form, or as a result of mutations or toxin application [22].

Our data and previous publications clearly demonstrate that hSGLT3 does not transport sugar, but it senses the sugar [3]. We do not know if the “channel-like” behavior of the sensor is actually a cation uniporter or a channel. However, whether hSGLT3 is a channel or a uniporter, to our knowledge the E457Q-hSGLT3 mutant is the first demonstration of the conversion of a protein with channel-like behavior into a coupled transporter in a well-defined stoichiometry. Thus, this mutant may provide a unique model in which to further investigate the biophysical differences between channels and transporters. In addition, since only a single amino acid change results in this altered function, our data suggest that the conformational changes involved in transport function are likely to be similar to those involved in glucose sensor function, comparable to what has been proposed for the differences in CLC channel/transporter function [23].

### E457Q-hSGLT3 sugar transport

We showed that by changing one amino acid, E457, in hSGLT3 for the amino acid present in hSGLT1, Q457, we converted this sugar sensor into a sugar transporter with the same stoichiometry as hSGLT1. Moreover, by replacing the analogous amino acid in hSGLT1 for glutamate (Q457E-hSGLT1) hSGLT1 became an uncoupled transporter (Fig. 2B). So, why is E457Q-hSGLT3 able to transport? Several studies indicate that sugars interact directly with the residue analogous to 457 in SGLT proteins [8], [9]. Sugars, although they are not transported by WT-hSGLT3, do bind to the protein and depolarize the membrane. This suggests that even though residue 457 is glutamate in WT-hSGLT3, it still likely interacts with sugars. However, the interaction between glutamate and sugar in hSGLT3 may be different than in SGLT1, where residue 457 is glutamine. Another possibility is that in hSGLT3, the conformational changes that in SGLT1 follow sugar binding and result in sugar transport may not occur due to the presence of glutamate at this position.

An alternative explanation for the presence of transport in E457Q-hSGLT3 is that the glutamine substitution enhances Na^+^ binding. This hypothesis is supported by data in Figure 3B and 3E that show greater presteady-state currents in the mutant protein than in the wild type. Pre-steady-state currents in SGLT proteins are thought to reflect conformational changes in the protein after Na^+^ binding has occurred, but before sugar binding. The pre-steady-state currents in E457Q-hSGLT3 are not large, but in all oocytes tested they were larger than in any WT-hSGLT3 expressing oocyte. Even though we cannot completely rule out that these larger pre-steady-state currents are due to a higher expression level of E457Q-hSGLT3 than WT, our results suggest that a glutamine at position 457 may promote Na^+^ binding to the protein, which could allow hSGLT3 to function as a transporter by enabling necessary conformational changes prior to sugar binding. Therefore, residue 457 may be essential for either binding sugars in a manner that allows their transport or for allowing the right conformational changes that either precede or follow sugar-binding to occur and that ultimately result in transport of sugars. These possibilities are not mutually exclusive, and may all apply. Future detailed biophysical experiments will allow discrimination between these and other potential explanations.

### Interaction of sugars with SGLT proteins

How do sugar specificity and K_0.5_s change in E457Q-hSGLT3? As seen in Voss et al. [4] and Table 1, most sugars tested have lower K_0.5_ for hSGLT1 than for hSGLT3, with imino sugars being the exception. Imino sugars have nitrogen instead of oxygen in their ring structure. The imino sugar DNJ interacts with hSGLT3 with a K_0.5_ almost 5,000 times lower than glucose (4 µM compared to 19 mM for glucose), and 50 µM DNJ induces depolarizations that are double those of glucose [4]. Conversely, hSGLT1 does not interact with DNJ [4]. In E457Q-hSGLT3, 500 µM DNJ induced a very small depolarization (∼5-fold smaller than glucose-induced depolarization), indicating that the interaction of the imino sugar DNJ with E457Q-hSGLT3 is much worse than with WT-hSGLT3. In the vSGLT crystal structure, the side-chain of the glutamine corresponding to Q457 interacts directly with the oxygen (O5) in the ring of the sugar molecule [9], as well as with the 6′-OH, suggesting that the poor interaction of DNJ with E457Q-hSGLT3 and hSGLT1 is due to the presence of a glutamine at position 457.

The removal of the 6′-OH group from glucose (6DO-glc) increases K_0.5_ in hSGLT1 6-fold, and in hSGLT3 the interaction with 6DO-glc is so poor that no K_0.5_ can be measured [4]. However, in E457Q-hSGLT3, the 6DO-glc K_0.5_ was similar to that of hSGLT1. Our data might be interpreted to suggest that the interaction with 6DO-glc is favored if the amino acid at this position is glutamine instead of glutamate. However, pig SGLT3, which has glutamate at position 457, interacts with 6DO-glc even more strongly than with glucose [8]. Thus, while the side chain is likely to play a role, determinants elsewhere in the binding site of SGLT proteins cannot be discounted in determining the sugar specificity.

The lack of a -OH group at positions 2 (2DO-glc) or 3 (3DO-glc) causes loss of sugar binding in both hSGLT1 and hSGLT3 [4]. Similarly, E457Q-hSGLT3 did not interact with 2DO-glc. However, it did interact reasonably well with 3DO-glc (K_0.5_ = 2.2 mM) displaying a maximal current similar to glucose. Given that the side chain of 457 is likely to contact the opposite end of the sugar, our data suggest that the E457Q mutation leads to allosteric changes in the binding site that permit interaction with 3DO-glc.

Finally, the change in the orientation of the 4′-OH group in galactose with respect to glucose does not change the interaction of sugar with hSGLT1; however it does in WT-hSGLT3 to the point that we see no interaction with galactose [3]. Galactose-induced depolarization in E457Q-hSGLT3 expressing oocytes, but the K_0.5_ was 134 mM. This K_0.5_ is more than 400 times weaker than the K_0.5_ of glucose (Ratio^glc^ = 447, Table 1). Therefore, we conclude that the mutation had no favorable effect on the discrimination between glucose and galactose.

Overall, we observed that the K_0.5_s of sugars for E457Q-hSGLT3 resembled those of hSGLT1 more than hSGLT3, along with changes in specificity, namely an increased selectivity against imino sugars and for 3DO-glc, indicating the remarkable role of amino acid 457 in determining both binding affinity and specificity in SGLT proteins.

### Physiological glucose sensing

While all cells of the body use glucose as a source of energy, some cell types detect the amount of glucose present in the extracellular environment by proportionally depolarizing the membrane to the sugar concentration. This is termed glucose sensing and has been described in pancreas [24], portal vein [25], brain [26], [27] and carotid body [28], among other tissues. A well-described glucose sensing mechanism involves the sugar transporter GLUT. GLUT transports glucose into the cell, where it is metabolized through glycolysis. Glycolysis increases the ATP levels, thus inhibiting ATP-sensitive K^+^ channels, and leading to depolarization of the plasma membrane. Human SGLT3 has also been proposed to function as a glucose sensor after being characterized by expression in *Xenopus laevis* oocytes where it was found to depolarize the membrane in response to extracellular glucose, without any sugar transport [3].

Since that first report, several studies have suggested that SGLT3 may have sugar sensing functions in different tissues. In the gastrointestinal system, Freeman et al. [5] proposed that detection of glucose in the intestine that subsequently inhibits gastric emptying and stimulates intestinal fluid secretion involves SGLT3. In the brain, O'Malley et al. [6] suggested that glucose-excited neurons can sense glucose through the actions of SGLT proteins, and detected SGLT3, among other SGLTs, in rat hypothalamic primary cultures. However, the glucose sensing property of hSGLT3 *in vivo* has not been tested until now. In this work we show, by expressing hSGLT3 in *C. elegans* ASK chemosensory neurons, that hSGLT3 can function as a sugar sensor *in vivo*. We show that *C. elegans* that express WT-hSGLT3 in those cells are repulsed by or attracted to glucose depending on the pH. We also show that the attraction is inhibited by phlorizin. Interestingly, *C. elegans* expressing E457Q-hSGLT3 are repulsed by glucose at pH 6 and do not show any preference for glucose over the control spot at pH 5. Wild type control *C. elegans* are neither repulsed by nor attracted to 10 mM glucose at either pH (Fig. 5). One explanation of why *C. elegans* that express WT-hSGLT3 are repulsed by glucose on standard chemotaxis plates (pH 6) but are attracted to this sugar at pH 5 could be that ASK sensory neurons are capable of mediating both attraction and repulsion [13]–[15] and the larger H^+^ concentration at pH 5 can exert an effect on the behavior. The magnitude of the WT-hSGLT3 glucose-induced currents cannot be directly compared with E457Q-hSGLT3's because they depend on their level of expression in oocytes and in *C. elegans*. However, we can compare how WT-hSGLT3 and E457Q-hSGLT3, expressed in oocytes, respond to glucose at those pHs. As seen in Figure 6A, in WT-hSGLT3 expressing oocytes there is a current when the pH is lowered from 6 to 5, and 10 mM glucose induces small currents at pH 6, but much larger currents at pH 5. It is possible that small currents mediated by WT-hSGLT3 on standard plates (pH 6) result in repulsion and that larger currents that occur on pH 5 plates result in attraction. Alternatively, at different pHs different cations could carry the current, thus leading to different behavioral outcomes; thus at pH 5, H^+^ ions carry more current than Na^+^ in WT-hSGLT3. Viewed strictly as a depolarization, different cations would not be expected to have different effects; however the translocation of H^+^ into the cell at pH 5 could have a different effect than Na^+^ on *C. elegans* neuronal physiology due to a potential change in internal pH. This could also explain why E457Q-hSGLT3, whose glucose-induced currents are less pH sensitive (Fig. 6B) than WT-hSGLT3 (which suggests that the amount of H^+^ transported is less), mediates repulsion on standard plates but shows no preference at pH 5. The idea that less H^+^ are transported in E457Q-hSGLT3 is supported by the calculation of the E_rev_, that indicates that H^+^ permeate less through the mutant protein. Regardless of the precise mechanism by which WT-hSGLT3 and E457Q-hSGLT3 affects *C. elegans* neuronal physiology, which is beyond the scope of this work, our data demonstrate that hSGLT3 is capable of acting as a *bona fide* glucose sensor *in vivo*, and that its action is sufficient to result in altered behavior in the context of a living organism.

## Materials and Methods

### Mutagenesis

The plasmid containing WT-hSGLT3 cDNA was used as a template for site-directed mutagenesis. Glutamate at amino acid 457 was replaced by glutamine (E457Q) using the QuikChange kit (Stratagene). The oligonucleotide primers used were: sense, 5′- ATCCATTACACA**C**AATCAATTTCTAGC -3′; and antisense, 5′- GCTAGAAATTGATT**G**TGTGTAATGGAT -3′. Bold letters represent the nucleotides changed. The gene was sequenced from start codon to stop codon to ensure that only the desired mutation was present. For information about Q457E-hSGLT1 construct see reference [8].

### Expression of proteins in *Xenopus laevis* oocytes

WT-hSGLT3 and E457Q-hSGLT3 cDNAs were linearized with XbaI and RNA was transcribed and capped *in vitro* using the T3 RNA promoter (MEGAscript kit, Ambion). Mature *Xenopus laevis* oocytes were injected with 50 ng of cRNA coding for each protein. Oocytes were maintained in OR2 supplemented with penicillin (10,000 U/ml)/streptomycin (10 mg/ml) at 19°C for 3–8 days until used.

### Electrophysiology

Experiments were performed at room temperature using the two-electrode voltage-clamp method in a rapid perfusion chamber to measure changes in membrane potential. For the experiments, the oocytes were bathed in Na^+^ buffer composed of (mM): 100 NaCl, 2 KCl, 1 MgCl_2_, 1 CaCl_2_, 10 Hepes/Tris, pH 7.4, and in Na^+^-free buffer where choline-Cl replaced NaCl. The pH 5.0 solutions contained the same ions and used MES as a buffer. The experiments were controlled and data were acquired using pClamp software (Axon Instruments). The apparent sugar affinities (K_0.5_) were obtained by measuring depolarizations in oocytes with increasing sugar concentrations (from 0 to 150 mM). The depolarizations were fit to equation
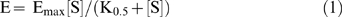
where E_max_ is the maximal depolarization, [S] is the substrate concentration, and K_0.5_ is the substrate concentration for 0.5 E_max_.

### Transport experiments

αM-glc transport into oocytes was measured using a radioactive tracer technique [29]. The oocytes were incubated in 50 µM αM-glc with traces of ^14^C-αM-glc for 1 hour in Na^+^ buffer at pH 7.5. The oocytes were then washed with cold Na^+^-free solution, and individually solubilized with 10% SDS. Sugar uptake was determined by using a scintillation counter. Sugar uptake in non-injected oocytes from the same batch of oocytes was used as control.

For experiments studying the stoichiometry, E457Q-hSGLT3 oocytes were clamped at -80 mV and when base line current was stable, the oocyte was perfused with 1 mM αM-glc (2 mM αM-glc in Q457E-hSGLT1) containing trace ^14^C-αM-glc for several minutes. The presence of sugar induced current in E457Q-hSGLT3 or Q457E-hSGLT1 expressing oocytes. The sugar was then removed from the bath and induced current returned to the base line. The oocyte was then washed, solubilized and counted as described. The induced current was converted to its molar equivalent as described before [30].

### Molecular biology in *C. elegans*

The *Psrg-8:hSGLT3-GFP* vector was created by introducing WT-hSGLT3 or E457Q-hSGLT3 cDNA without stop codon in frame with GFP into vector pPD95.77 which includes enhanced GFP (constructed by Scott Clark, NYU) and in which we previously introduced the promoter of the *srg-8* gene [16]. Prior to subcloning into pPD95.77, WT-hSGLT3 or E457Q-hSGLT3 cDNA and *srg-8* promoter were cloned into TOPO pCR2.1 vector for amplification and sequencing.

### *C. elegans* strains and growth

Nematode strains were maintained at 20°C on standard nematode growth medium (NGM) seeded with *Escherichia coli* strain OP50 as food source [31]. WT-hSGLT3 or E457Q-hSGLT3 plasmids were injected into wild type N2 *C. elegans*, cotransformation marker was pPD117.01 vector which encodes GFP under the control of the promoter of the *mec-7* gene expressed in mechanosensory neurons [32].

### Behavioral assays

To test chemotaxis to glucose and lysine-acetate we followed standard procedures [13]. Briefly, a chunk of agar 1 cm in diameter was removed from 10 cm plates and soaked in the test solution (10 mM glucose, 10 mM glucose with phlorizin, or 0.5 M lysine-acetate) for 3 h. Chunks were put back in the plate overnight to allow equilibration and formation of a gradient. When we tested the effect of phlorizin, we incubated the chunk of agar in 10 mM glucose plus 0.1 mM phlorizin prior to re-inserting the chunk back into the plate. Thirty to forty *C. elegans* were then placed between the glucose spot and a control spot on the opposite side of the plate (Fig. 5A). 10 µL of 20 mM NaN_3_ was placed on both spots to anaesthetize animals once they reached the spot. After 1 hour animals on each side of the plate were counted and attraction index (AI) was determined as follows: (number of animals at glucose spot - number of animals at control spot)/(total number of animals). Control spot is the place on the plate at the opposite side of glucose spot. An AI of 1.0 indicates complete attraction; an AI of 0 indicates a random distribution of the animals on the assay plate; a negative AI is indicative of repulsion.

## Supporting Information

Figure S1 Recording of currents in a non-injected oocyte. A. Currents recordings in a non-injected oocyte perfused with the Na^+^ solution. Voltage pulses were the same as shown in Figure 3A. B. The same oocyte was perfused with the Na^+^ solution plus 150 mM αM-glc. C. Current-voltage relationships of currents shown in A and B.
(0.23 MB TIF)

Figure S2 WT-hSGLT3 does not transport sugar in a low pH solution. αM-glc uptake in oocytes expressing hSGLT3 compared with non-injected oocytes at pH 5 for 1 hour. The data show that hSGLT3 oocytes did not transport sugar at this pH.
(0.11 MB TIF)
