# Supplementary figures and images for: A Single Amino Acid Change Converts the Sugar Sensor SGLT3 into a Sugar Transporter
Source: PLoS One. 2010 Apr 20;5(4):e10241. doi: 10.1371/journal.pone.0010241 (PMC2857651; doi:10.1371/journal.pone.0010241)

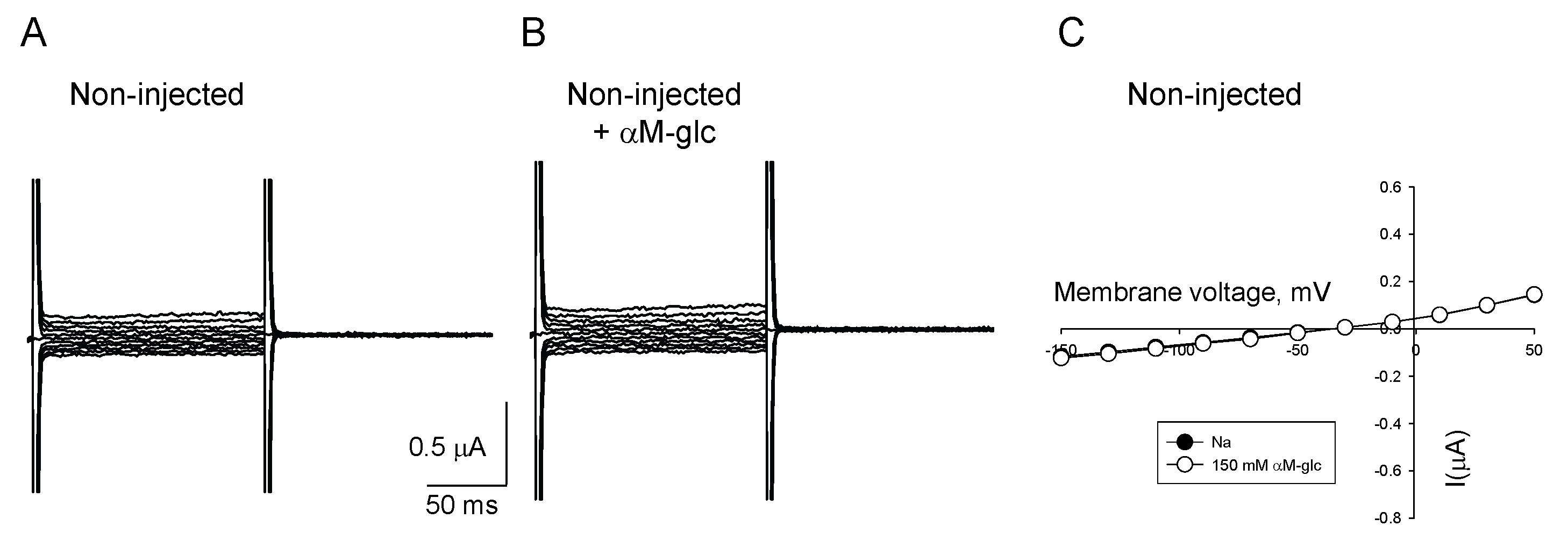

Supplement: Figure S1 — Recording of currents in a non-injected oocyte. A. Currents recordings in a non-injected oocyte perfused with the Na+ solution. Voltage pulses were the same as shown in Figure 3A. B. The same oocyte was perfused with the Na+ solution plus 150 mM αM-glc. C. Current-voltage relationships of currents shown in A and B. (0.23 MB TIF) [file pone.0010241.s001.tif]

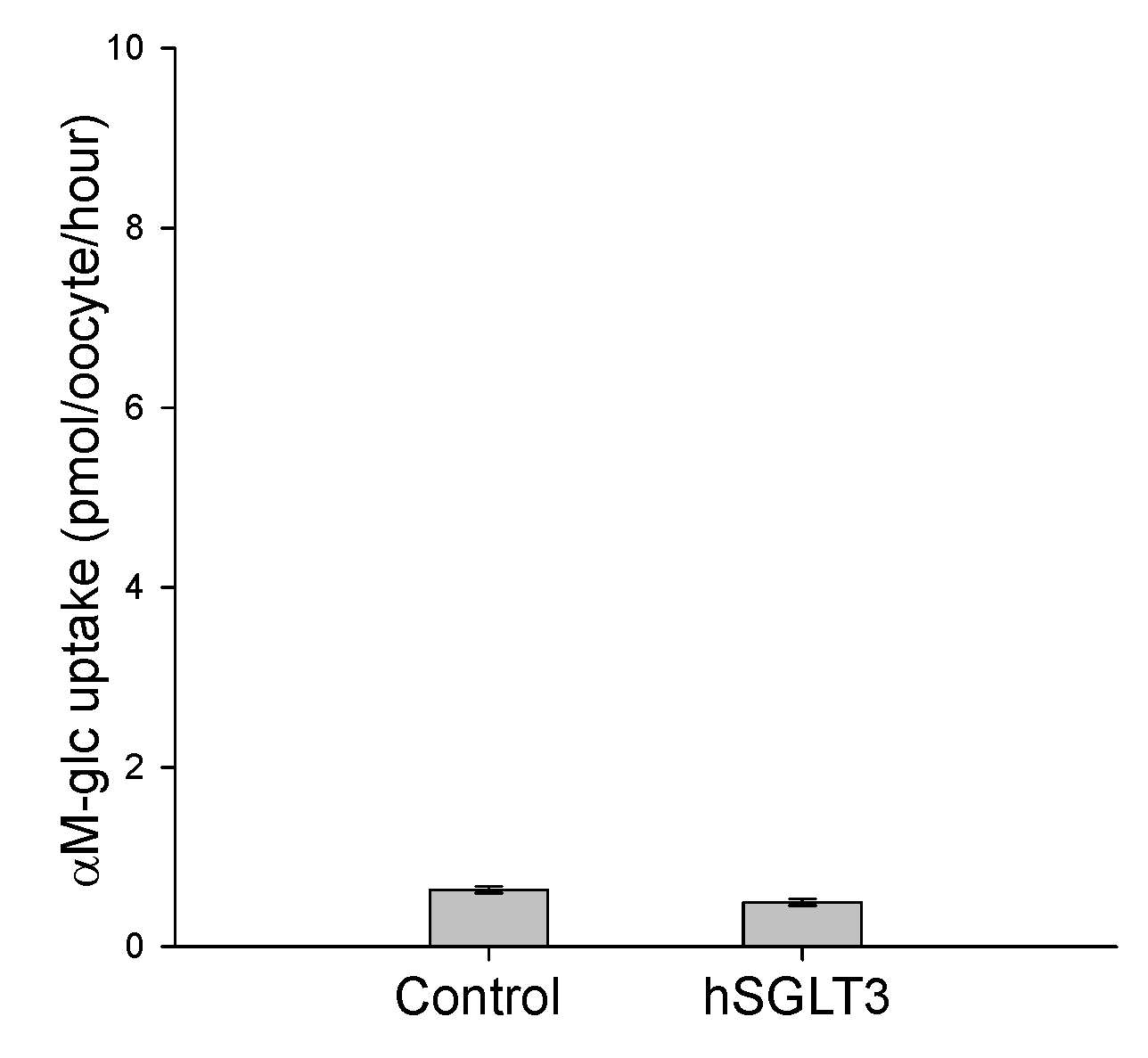

Supplement: Figure S2 — WT-hSGLT3 does not transport sugar in a low pH solution. αM-glc uptake in oocytes expressing hSGLT3 compared with non-injected oocytes at pH 5 for 1 hour. The data show that hSGLT3 oocytes did not transport sugar at this pH. (0.11 MB TIF) [file pone.0010241.s002.tif]
